# Spinosad resistance in field populations of melon fly, *Zeugodacus cucurbitae* (Coquillett), in Hawaii

**DOI:** 10.1002/ps.6583

**Published:** 2021-08-13

**Authors:** Ju‐Chun Hsu, Ming‐Yi Chou, Ronald FL Mau, Colby Maeda, Ikkei Shikano, Nicholas C Manoukis, Roger I Vargas

**Affiliations:** ^1^ Department of Entomology National Taiwan University Taipei Taiwan; ^2^ Agricultural Extension Center National Chung Hsing University Taichung Taiwan; ^3^ Department of Plant and Environmental Protection Sciences University of Hawai'i Mānoa HI USA; ^4^ US Department of Agriculture, Agricultural Research Service Daniel K. Inouye US Pacific Basin Agricultural Research Center Hilo HI USA

**Keywords:** Cucurbitaceae fruits, GF‐120, insecticide resistance, integrated pest management, protein bait

## Abstract

**BACKGROUND:**

Control of *Zeugodacus cucurbitae*, a serious agricultural pest worldwide, often includes or is dependent on the use of spinosad‐based insecticides. This is especially the case in Hawaii, where GF‐120, a protein bait containing spinosad as the active ingredient, has been in use as a key integrated pest management (IPM) tool against this Tephritid for the last two decades. Here, we report on resistance to spinosad [resistance ratios (RRs) and median lethal concentration (LC_50_)] in Hawaii's populations of *Z. cucurbitae*.

**RESULTS:**

High resistance was found in populations from three farms on Oahu (RR = 102–303; LC_50_ = 191–567 mg L^–1^) and in a population from Maui (RR = 8.50; LC_50_ = 15.9 mg L^–1^). These will be problematic for control given that the most concentrated dilution ratio on the GF‐120 label is 96 mg L^–1^ of spinosad (1 part GF‐120 to 1.5 parts water). Background resistance in a naïve wild population from the Island of Hawaii (RR = 2.73; LC_50_ = 5.1 mg L^–1^) was relatively low compared with a spinosad‐susceptible laboratory colony (LC_50_ = 1.87 mg L^–1^). Resistance in the three Oahu and one Maui populations declined over generations in the absence of spinosad but remained elevated in some cases. Moreover, melon flies collected from one of the Oahu farms 1 year after the cessation of spinosad use revealed high persistence of resistance.

**CONCLUSION:**

Compared with a 2008 survey of spinosad resistance, our findings indicate a 34‐fold increase in resistance on one of the Oahu farms over 9 years. The evolution and persistence of high levels of resistance to spinosad in *Z. cucurbitae* in Hawaii highlights the need for alternative control tactics, particularly rotation of active ingredients. © 2021 The Authors. *Pest Management Science* published by John Wiley & Sons Ltd on behalf of Society of Chemical Industry.

## Graphical abstract

Frequent and repeated use of protein bait containing spinosad (GF‐120) in Hawaii has resulted in high levels of spinosad resistance in field populations of melon flies, which can persist for multiple generations.

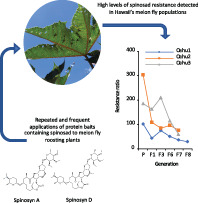

## 1 INTRODUCTION

The melon fly, *Zeugodacus cucurbitae* (Coquillett) (Family: Tephritidae), is a devastating pest of plants in the family Cucurbitaceae. While cucurbits are the preferred hosts of *Z. cucurbitae*, its reported host range is much wider,^1^ and thought to include at least 136 plant taxa across 62 genera.^2^ While melon fly is considered to be native to southeast Asia, it has successfully invaded many temperate, tropical, and sub‐tropical regions of the world.^1^ *Zeugodacus cucurbitae* was first discovered on the Hawaiian Islands in 1895,^3^ and has since become the most important pest of cucurbit crops in Hawaii. It is also known to infest ripe fruits of the introduced papaya (*Carica papaya*) tree in Hawaii.^4^

Female *Z. cucurbitae* prefer to lay eggs soon after fruit set, while the skin of the fruit is still soft.^1^ Other than during oviposition, *Z. cucurbitae* spend most of their time on non‐host plants, usually in the perimeter of the host crop fields.^5^ They are well‐documented to aggregate on these ‘roosting’ plants.^6^ Therefore, management practices in Hawaii have changed from full coverage crop sprays to targeted insecticide and protein bait treatments on the roosting plants, which are either naturally occurring or planted around the perimeter of the crop fields.^5^

In 2001, the protein bait GF‐120, containing spinosad as the active ingredient, was introduced and applied as targeted applications to roosting plants of *Z. cucurbitae*.^7^, ^8^, ^9^ Spinosad has a unique mode of action, serving as both contact and stomach poisons.^10^ This insecticide is also selective in that it has particularly high activity against certain pest insect groups including moths, thrips and tephritid flies, but has low risk to the environment and not‐target animals.^10^, ^11^ In 2002, GF‐120 was employed as a key component of an area‐wide fruit fly pest management (HAW‐FLYPM) program to control *Z. cucurbitae* populations in Hawaii.^12^

Hawaii's mild climate allows for year‐round production of cucurbit crops. Due to local market demand, many small producers specialize on a particular crop, resulting in adjacent, successive plantings of cucurbits. These optimal conditions allow *Z. cucurbitae* to produce more than ten generations per year.^13^, ^14^ Continued year‐round use of GF‐120 since 2002 has subjected the *Z. cucurbitae* populations to intensive selection pressure, which raises significant concern for the evolution of resistance to the active ingredient spinosad. Evolved resistance to spinosad has been reported in other Tephritidae species.^15^, ^16^, ^17^, ^18^Thus, Hsu *et al*.^19^ surveyed for spinosad resistance in *Z. cucurbitae* field populations in August 2008 and found low levels of resistance in populations collected from Pingtung, Taiwan [resistance ratio (RR) = 13.3] and Ewa, Hawaii (RR = 15.5). More recently in 2015, cucurbit producers in Oahu reported failed efforts to control *Z. cucurbitae* populations using GF‐120 (Ronald Mau, personal communications). A simple bioassay at the field site suggested that spinosad resistance was likely.

Therefore, the present study was conducted to examine the prevalence and strength of spinosad resistance in field populations of *Z. cucurbitae* in Hawaii. Given the continued use of GF‐120 since the last assessment for resistance in 2008, we hypothesized that spinosad resistance has increased in *Z. cucurbitae* populations. We further examined whether the resistant trait had become a fixed allele in the spinosad‐resistant populations or the levels of resistance would decline after multiple generations of no exposure to spinosad.

## 2 MATERIALS AND METHODS

### 2.1 *Zeugodacus cucurbitae* field populations

In June and July 2017, field populations of *Z. cucurbitae* were collected as eggs and larvae from three farms on Oahu from infested watermelon (site name: ‘Oahu 1’), honeydew melon (‘Oahu 2’) and cucumber (‘Oahu 3’) crops. A fourth field population was collected from infested zucchini on Maui (‘Maui’), and a fifth population was collected from infested papaya on Hawaii Island (‘Wild’) (Table 1). We called this fifth population ‘Wild’ because it was collected from a papaya farm where, to the best of our knowledge, GF‐120 had never been used, compared to cucurbit farms with heavy usage of GF‐120. The infested fruit from Oahu and Maui were held at 2.8 °C for up to 2 days prior to being air shipped to the US Department of Agriculture Agricultural Research Service (USDA ARS) Daniel K. Inouye Pacific Basin Agricultural Research Center. Upon delivery, the infested fruits were kept in individual containers and supplemented with the same original host fruits as well as dry larval diet (1585 g wheat mill feed, 718 g white granulated sugar, 200 g torula yeast, 6.7 g nipagin, 5.6 g sodium benzoate). The container was placed inside a holding box (‘Lewis box’) with a thin layer of sand on the bottom to allow pupation.^20^ The number of pupae obtained from each field site was counted (Table 1). Subsequent generations were reared on ripe papaya (*C. papaya*) fruit with dry larval diet at 22 ± 1 °C, 55 ± 6% relative humidity (RH) and 12 h:12 h photoperiod without exposure to spinosad or any other insecticide. The Oahu and Maui field collected populations were maintained at the USDA ARS Daniel K. Inouye Pacific Basin Agricultural Research Laboratory for up to 12 generations (F12), and the Hawaii Island population for three generations (F3).

**Table 1:** *Zeugodacus cucurbitae* populations collected for spinosad resistance assessment

Population name	Host fruits	Location^1^	Latitude (N)	Longitude (W)	Number of pupae collected
Oahu 1^a^	Watermelon	Poamoho (Oahu)	21°31′	158°09′	6782
Oahu 2^a^	Honeydew	Ewa (Oahu)	21°20′	158°03′	1255
Oahu 3^a^	Cucumber	Ewa (Oahu)	21°20′	158°02′	1951
Maui	Zucchini	Kula (Maui)	20°79′	156°36′	5683
Wild^b^	Papaya	Keaau (Hawaii)	19°63′	155°07′	Multiple collections
Oahu 4^c^	Cucumber	Poamoho (Oahu)	21°55′	158°09′	1248

^a^ Oahu 1–Oahu 3 populations were collected in June and July 2017.

^b^ Collected infested papaya about 3–4 weeks before each trial. Infested papaya fruits were collected multiple times throughout the year.

^c^ Oahu 4 population was collected in September 2018, approximately 1 year after farmers ceased use of GF‐120. Oahu 1 farm is in close proximity to Oahu 4 (< 500 m), thus likely comprising of the same melon fly population.

Farmers on Oahu and Maui ceased the use of GF‐120 in September 2017 due to the lack of efficacy and recommendations from the University of Hawai'i, College of Tropical Agriculture and Human Resources, Cooperative Extension. Therefore, in September 2018, a sixth population (Oahu 4) was collected from a farm that was in close proximity to the Oahu 1 population collection site (< 500 m apart). The Oahu 4 population was used to assess if resistance had naturally faded following the cessation of GF‐120 application (Table 1).

A spinosad‐susceptible laboratory line of *Z*. *cucurbitae*, which had been maintained for over 500 generations without exposure to insecticides, was used as a control. The spinosad‐susceptible line was reared on an artificial larval diet and maintained under controlled environmental conditions of 25 ± 1 °C and 68 ± 3% RH following standard protocol.^21^ Pupae were placed in screen cages (27 cm × 27 cm × 27 cm) and emerging adults were supplied with water and a mixture of one part hydrolyzed yeast (MP Biomedicals, Solon, OH, USA) and three parts sugar.

### 2.2 Preparation of spinosad concentrations

A stock solution of 10 000 μg mL^–1^ active ingredient (spinosad) was produced by mixing 445 ± 0.4 mg of Entrust SC Naturalyte Insect Control (spinosad 22.5%; Dow AgroSciences LLC., Indianapolis, IN, USA) with 10 mL of distilled water. The solution was then serially diluted to produce concentrations ranging from 2000 to 0.31 μg mL^–1^ in a dietary solution containing 20% sugar and 5% peptone (Fisher Bio Reagents, Waltham, MA, USA). The dietary solution without Entrust SC was used as a control.

For the diagnostic test of resistance, a single concentration of GF‐120 NF Naturalyte Fruit Fly Bait (Dow AgroSciences LLC.) was produced according to the product label (1 part GF‐120 to 1.5 parts distilled water) by mixing 100 mL of GF‐120 with 150 mL of distilled water to produce a final volume of 250 mL. The GF‐120 used in this study contained 0.02% spinosad (*w/w*), which equates to 0.002 lb of spinosad per gallon (0.24 g L^−1^). Therefore, the final diluted concentration contained 96 mg L^–1^ spinosad (i.e. 96 ppm spinosad). GF‐120 Blank Adjuvant Mix SL (spinosad 0%) (Dow AgroSciences LLC.) served as the spinosad‐free control, and was also diluted 1:1.5 distilled water.

### 2.3 Spinosad resistance assays

The feeding application assay was modified from Hsu and Feng^15^ and Chou *et al*.^22^ Dental cotton rolls (Pearson, No.2 medium 3.8 cm × 0.95 cm) were cut in half and treated with approximately 0.4 mL of GF‐120 or GF‐120 Blank for the diagnostic test and 0.4 mL of a spinosad concentration or a control to assess spinosad resistance. Wicks were inserted through a 1‐cm cross‐slit in the cover of 355 mL clear, plastic, deli containers. Twenty flies were exposed to the treated wicks for 24 h, starting in the early afternoon (approximately 1 p.m.). After this period the treated wicks were replaced with diet‐only wicks, which were exchanged daily. Prior to each trial, flies were starved for 24 h to facilitate feeding in the bioassay (except the parental generation). Cups were maintained in the laboratory (24 ± 1 °C, 50 ± 5% RH, 12 h:12 h photoperiod), and mortality was recorded at 72 h after placement in the treatment cups. Our definition of mortality included flies that were dead and those that were still alive but could no longer stand. Two cups containing 20 flies from each population were set up at the same time for each treatment, totaling 40 flies per treatment.

Parental and F3 generation flies used in the assays were less than 23 days post‐emergence, while all other generations were assayed at less than 10 days post‐emergence. On average, female and male adult melon flies maintained under our laboratory conditions (24°C) survive for 79.1 and 136.6 days, respectively.^23^ For the ‘Wild’ population, only the P and F1 generations were tested for resistance to GF‐120 and P, F1 and F3 generations for spinosad resistance. P, F1, F3, F6 and F8 generations were tested for the Oahu 1–Oahu 3 populations, except for Oahu 2 which was not tested in the F8 generation. The Maui population was tested in the P, F1, and F6 generations. Only the parental generation was tested for the Oahu 4 population. Mortality of the spinosad‐susceptible laboratory population was used to control for mortality levels.

### 2.4 Data analyses

The post‐treatment mortality data for concentration‐response spinosad resistance assays were subjected to probit analyses with PoloPlus software (LeOra Software LLC, Parma, MO, USA) to obtain the median lethal concentration (LC_50_). Spinosad concentrations with 100% and 0% mortality were dropped from the data analysis. The *χ* ^2^ values were used to assess how well the individual LC_50_ values observed in the bioassays agreed with the calculated linear regression lines (goodness‐of‐fit).^24^ The RR was calculated by the LC_50_ value of the wild population against the value of the spinosad‐susceptible laboratory line. No analyses were conducted for the single concentration GF‐120 resistance assays.

## 3 RESULTS

Melon flies that emerged from field‐collected fruits exhibited lower mortality (≤ 40% mortality) after exposure to GF‐120 than the laboratory population after 72 h (100% mortality; Table 2), except for the wild population (85% mortality) that was collected from a papaya farm where GF‐120 had not been used. Susceptibility to GF‐120 increased in subsequent generations, but the populations varied in how quickly this occurred. The F1 generation of the Oahu 2 and Maui populations exhibited higher susceptibility to GF‐120 (72.5 and 82.5% mortality, respectively) than the parental generation, but mortality of the Oahu 1 and Oahu 3 populations in the F3 generation remained low (25 and 35% mortality, respectively). Exposure to GF‐120 killed 100% of the Maui population by the F6 generation. However, GF‐120 exposure did not kill 100% of any of the Oahu populations up to the F8 generation.

**Table 2:** Percent mortality of field‐collected melon fly populations and a laboratory colony 72 h after exposure to GF‐120

Population	Mortality (%) 72 h after exposure to GF120 (1:1.5 distilled water)					
	P	F1	F3	F6	F7	F8
Laboratory^a^	100	100	100	100	97.5	100
Wild	82.5	97.5	—	—	—	—
Oahu 1	15	—	25	67.5	90	85
Oahu 2	12.5	72.5	65	82.5	72.5	—
Oahu 3	15	47.5	35	82.5	70	70
Maui	40	82.5	67.5	100	—	—

The parental generation (P) were obtained from field‐collected fruits. The following generations (F1–F8) were maintained in the laboratory and had no exposure to GF‐120 or spinosad.

^a^ The generations since field collection does not apply to the laboratory population, which was only used to assess resistance of the field collected flies.

When the field‐collected Wild melon fly population from Hawaii Island was exposed to concentration ranges of spinosad, they exhibited an LC_50_ value of 5.1 μg mL^–1^ (*χ* ^2^ = 3.11, DF = 7, slope = 3.03) and a resistance ratio of 2.73 at 72 h after spinosad exposure (Table 3). Since this population had never been exposed to GF‐120, this level of resistance to spinosad is likely within the range of natural susceptibility. The parental generation of the Maui population had a higher LC_50_ (15.9 μg mL^–1^; *χ* ^2^ = 12.6, DF = 5, slope = 1.43) and RR (RR = 8.50) than the Wild population, though the 95% fiducial limits of the LC_50_ of the two populations overlapped (Table 3). The resistance of the Maui population declined rapidly and was similar to that of the laboratory population by the F6 generation (RR = 1.14) (Table 3).

**Table 3:** The median lethal concentration (LC_50_, μg mL^–1^) and resistance ratios (RRs) of successive generations [parental (P) to eighth generation (F8)] of several field‐collected melon fly populations to spinosad 72 h post‐treatment

		Generations since field collection					
Populations	Measure	P	F1	F3	F6	F7	F8
Laboratory^a^	LC_50_ (95% FL^b^)	1.87 (0.63–4.60)	1.87 (0.81–3.23)	2.34 (1.91–2.76)	3.48 (2.09–7.25)	5.93 (5.02–6.92)	3.79 (2.83–4.97)
Wild	LC_50_ (95% FL^b^)	5.10 (4.11–6.03)	3.81(2.28–5.82)	1.87(0.63–4.6)	—	—	—
	RR	2.73	2.04	0.80	—	—	—
Oahu 1	LC_50_ (95% FL^b^)	191 (64.8–491)	103 (89.6–148)	176 (56–362)	117 (84.6–154)	214 (103–507)	111 (18.7–290)
	RR	102	55.1	75.2	33.6	36.1	29.3
Oahu 2	LC_50_ (95% FL^b^)	567 (16.0–1140)	255 (141–463)	199 (159–250)	224 (119–800)	453 (331–678)	—
	RR	303	136	85.0	64.4	76.4	—
Oahu 3	LC_50_ (95% FL^b^)	347 (60.3–1190)	382 (120–2270)	491 (19.9–13 440)	267 (139–444)	348 (260–487)	481 (309–782)
	RR	185	204	210	76.7	58.7	127
Oahu 4^c^	LC_50_ (95% FL^b^)	46.7 (26.3–72.2)	—	—	—	—	—
	RR	25.0	—	—	—	—	—
Maui	LC_50_ (95% FL^b^)	15.9 (3.87–31.2)	7.66 (6.11–9.38)	—	3.95 (2.58–6.01)	—	—
	RR	8.50	4.10	—	1.14	—	—

The melon fly populations were maintained under laboratory rearing conditions and not exposed to spinosad after field collection. The RRs were calculating by dividing the LC_50_ of the field‐collected populations by the LC_50_ of the laboratory colony.

^a^ Laboratory colony was used for comparison to determine the resistance of the field collected populations. Hence the generations since field collection does not apply.

^b^ FL, fiducial limit.

^c^ Oahu 4 population was collected approximately 1 year after farmers discontinued use of GF‐120.

As expected from the GF‐120 assays, the LC_50_ values of the Oahu populations were significantly higher than the laboratory population (i.e. no overlap of the 95% fiducial limits). The LC_50_ of the parental generations was 191 μg mL^–1^ (*χ* ^2^ = 52.7, DF = 7, slope = 1.19) for Oahu 1, 567 μg mL^–1^ (*χ* ^2^ = 24.5, DF = 5, slope = 2.44) for Oahu 2, and 347 μg mL^–1^ (*χ* ^2^ = 17.8, DF = 5, slope = 1.28) for Oahu 3. The RR values of the parental generations of the three Oahu populations ranged from 102 to 303. Resistance did not decrease substantially in the absence of spinosad for at least three generations (RR of F3 generation ranged from 75 to 210). While resistance gradually declined, even after seven generations the LC_50_ values of the three Oahu populations were significantly higher than the laboratory population (i.e. no overlap of fiducial limits) and RRs ranged from 36 to 77.

The Oahu 4 population, which was field collected approximately 1 year after farmers ceased use of spinosad, had a significantly lower LC_50_ value than the parental generations of the other three Oahu populations collected over a year earlier (Table 3) but still significantly higher than the laboratory population (RR = 25). Control mortality for all assays did not exceed 5%.

## 4 DISCUSSION

Spinosad resistance was evident in populations of melon flies collected from cucurbit farms on Oahu and Maui. Resistance in the Maui population was low (RR = 8.50) and declined rapidly in the absence of spinosad exposure. Interestingly, mortality of the Maui parental population to the GF‐120 dilution (96 ppm = 96 μg mL^–1^) was considerably lower (40%) than expected from the LC_50_ (15.9 μg mL^–1^) determined using Entrust SC (spinosad) in a dietary solution of 20% sucrose and 5% peptone. It is possible that the GF‐120 dilution had a higher concentration of nutrients (protein and sugar) than the dietary solution. Another tephritid fruit fly, the Queensland fruit fly (*Bactrocera tyroni*), is known to alter the volume of liquid diet consumed to regulate its intake of calories.^25^ Thus, the higher susceptibility of the melon flies to spinosad in the dietary solution may be attributable to a higher ingestion of the dietary solution compared to GF‐120, and consequently influenced the ingested dose of spinosad. Moreover, spinosad uptake could have been affected by the loss of attractiveness of GF‐120 over time. Revis *et al*.^26^ demonstrated that GF‐120 was 11 times less attractive to female melon flies 2 h after application compared to freshly applied GF‐120. These are important factors because while laboratory resistance assays using spinosad in dietary solutions are useful for revealing the levels of resistance in field populations relative to laboratory susceptible strains, the assays may underestimate the resistance frequencies to the actual products used in the field, such as GF‐120 or spinosad products (e.g. Entrust SC and Success) mixed with protein baits (e.g. Nu‐lure). Resistance in the Oahu populations was much higher (RR > 100) than the Maui population. While resistance in the Oahu populations also declined in the absence of spinosad, complete susceptibility was not evident after eight generations.

Slow loss of resistance to spinosad has been reported previously. In the western flower thrips, *Frankliniella occidentalis*, a laboratory‐selected spinosad resistant population consisting of 100% resistant individuals maintained resistance for up to 8 months without spinosad exposure.^27^ Similar stability of resistance to spinosad has been reported in highly resistant tobacco budworm, *Heliothis virescens*.^28^ Resistance in *F. occidentalis* declined rapidly in the first 2 months after the immigration of susceptible individuals into the population. However, resistance levels after that initial decline remained stable for the next 6 months.^27^ The stability of spinosad resistance in *F. occidentalis* suggests a low fitness cost associated with the resistance mechanism in the absence of the insecticide.^29^ In our field‐collected melon fly populations, the decline in resistance from the parental generation to the subsequent generations without immigration of susceptible individuals suggests that there are fitness costs associated with the resistance mechanism. Fitness costs associated with spinosad resistance have been demonstrated in spinosad‐resistant (RR = 23) Oriental fruit fly, *B. dorsalis*; these costs included higher mortality rate, delayed development, and reduced fecundity.^30^

In the field, fitness costs associated with resistance traits will likely favor the selection for susceptible individuals to a greater degree than in the laboratory. Additionally, melon flies are strong flyers, with single continuous flight bouts sometimes lasting more than 100 min,^31^, ^32^ suggesting that potential immigration of susceptible individuals from surrounding areas is likely. Thus, the combination of fitness costs associated with resistance and immigration of susceptible individuals suggests that resistance in the field can be expected to decline fairly rapidly.

At variance with the hypothesis of rapid resistance decline in the field, however, is that 1 year after the cessation of GF‐120 use resistance was detected in the Oahu 4 population (RR = 25). This could be because the assumptions about fitness costs in the field or availability of susceptible individuals and their movement were incorrect, or because spinosad‐containing products were still being used by some farmers without our knowledge. It is generally thought that populations take longer to return from resistant to susceptible than they do to develop resistance.^33^ This would suggest that resistance in the Oahu 4 population 1 year after putative cessation of insecticide application will re‐emerge significantly faster following the reintroduction of the insecticide.

A previous collection of *Z. cucurbitae* was made at the Oahu 3 site in 2008.^19^ The LC_50_ (95% fiducial limit) of those flies collected in 2008 was 10.09 (6.36–19.4) μg mL^–1^ at 72 h post‐oral spinosad exposure.^19^ The LC_50_ (95% fiducial limit) value of the flies collected in 2017 and analyzed in the present study was 347 (60–1187) μg mL^–1^, which indicates that resistance increased by approximately 34‐fold in 9 years. Importantly, the LC_50_ values of the three Oahu populations (191–567 μg mL^–1^) far exceeded the spinosad concentration in the dilution of GF‐120 used in the field (96 μg mL^–1^). High levels of resistance on Oahu, particularly at the Oahu 2 and Oahu 3 collection sites which were located only about 1 km apart, likely occurred because of operational practices at the Oahu 2 location. In 2015, the farmer at the Oahu 2 farm began mixing Success (active ingredient 22.8% spinosad) with GF‐120 to produce concentrations of 500 ppm (500 μg mL^–1^) of spinosad, which temporarily improved control of the flies (Mau, personal communications), but also likely led to increasing resistance of the melon flies.

Dramatic increases in resistance upon increases in selection doses of spinosad have been shown in laboratory experiments. Young *et al*.^34^ demonstrated in a laboratory selection experiment that low dose topical applications of spinosad induced 75 to 85% mortality in tobacco budworm (*H. virescens*) until the fifth generation when mortality declined. At that point, the dose of spinosad was gradually increased every generation until the 11th generation to obtain 70% mortality at each generation. While the measured resistance of the *H. virescens* population in the sixth generation was only 1.68 times that of the parental generation, by the 14th generation resistance was 1068 times that of the parental generation. Similar results have been demonstrated in laboratory‐selected Oriental fruit flies, which were only 3.54 times more resistant than the susceptible parental generation after four generations of spinosad selection but became more than 408 times more resistant by the eighth generation.^15^ Resistance of a population can increase rapidly as more individuals in the population become resistant. In thrips (*F. occidentalis*), a population consisting of 50% resistant individuals was 10–21 times more resistant than a fully susceptible population. However, resistance in the population increased to 125–430 times higher than the susceptible population when the proportion of resistant individuals was increased to 75%.^27^

As mentioned earlier, spinosad resistance in the field was still observed at Oahu 4 more than a year after GF‐120 use was halted. Thus, alternative control methods based on insecticide rotations with spinosad (or GF‐120) included are needed. In spinosad‐resistant *Spodoptera exigua* (RR = 345), no cross‐resistance between spinosad and fenvalerate, phoxim, methomyl, abamectin, and cyfuthrin was detected.^35^ Furthermore, susceptibility of the spinosad‐resistant *S. exigua* to spinosad was increased by 9.8 times with the addition of the insecticide synergist piperonyl butoxide.^35^

In Hawaii, aside from spinosad, there are currently only two registered active ingredients (product names) for use on melon flies, naled (Diabrom 8 Emulsive and Diabrom Concentrate) and fipronil (Amulet C‐L). To operate an effective resistance management program, additional effective products in other insecticide classes are needed for use in a rotation. Numerous other classes of insecticides have demonstrated efficacy in other tephritid species^36^, ^37^, ^38^, ^39^ and we have found several insecticides, including beta‐cypermethrin, cyantraniliprole, abamectin, malathion and lamda‐cyhalothrin, to be effective against melon flies (Hsu *et al*., manuscript in preparation). An area‐wide approach consisting of insecticide rotations, lures, traps, cultural control methods, such as sanitation, and biological control agents are needed to provide consistent suppression of melon fly populations.^12^, ^40^ In conclusion, our findings reveal the evolution and persistence of resistance of melon fly populations in Hawaii to spinosad and highlight the need for alternative control tactics.
